# Functional Coordination of the Chromatin-Remodeling Factor AtINO80 and the Histone Chaperones NRP1/2 in Inflorescence Meristem and Root Apical Meristem

**DOI:** 10.3389/fpls.2019.00115

**Published:** 2019-02-07

**Authors:** Huijia Kang, Jing Ma, Di Wu, Wen-Hui Shen, Yan Zhu

**Affiliations:** ^1^State Key Laboratory of Genetic Engineering, Collaborative Innovation Center for Genetics and Development, International Associated Laboratory of CNRS-Fudan-HUNAU on Plant Epigenome Research, Department of Biochemistry, Institute of Plant Biology, School of Life Sciences, Fudan University, Shanghai, China; ^2^CNRS, IBMP UPR 2357, Université de Strasbourg, Strasbourg, France

**Keywords:** *Arabidopsis thaliana*, inflorescence meristem, root apical meristem, chromatin-remodeling factor, histone chaperone

## Abstract

Chromatin structure requires proper modulation in face of transcriptional reprogramming in the context of organism growth and development. Chromatin-remodeling factors and histone chaperones are considered to intrinsically possess abilities to remodel chromatin structure in single or in combination. Our previous study revealed the functional synergy between the Arabidopsis chromatin-remodeling factor INOSITOL AUXOTROPHY 80 (AtINO80) and the histone chaperone NAP1-RELATED PROTEIN 1 (NRP1) and NRP2 in somatic homologous recombination, one crucial pathway involved in repairing DNA double strand breaks. Here, we report genetic interplay between *AtINO80* and *NRP1/2* in regulating inflorescence meristem (IM) and root apical meristem (RAM) activities. The triple mutant *atino80-5 m56-1* depleting of both AtINO80 (*atino80-5*) and NRP1/2 (*m56-1*) showed abnormal positioning pattern of floral primordia and enlargement of IM size. Higher mRNA levels of several genes involved in auxin pathway (e.g., *PIN1*, *FIL*) were found in the inflorescences of the triple mutant but barely in those of the single mutant *atino80-5* or the double mutant *m56-1*. In particular, the depletion of AtINO80 and NRP1/2 decreased histone H3 levels within the chromatin regions of *PIN1*, which encodes an important auxin efflux carrier. Moreover, the triple mutant displayed a severe short-root phenotype with higher sensitivity to auxin transport inhibitor NPA. Unusual high level of cell death was also found in triple mutant root tips, accompanied by double-strand break damages revealed by γ-H2A.X loci and cortex cell enlargement. Collectively, our study provides novel insight into the functional coordination of the two epigenetic factors AtINO80 and NRP1/2 in apical meristems during plant growth and development.

## Introduction

Plant growth and development depend on a steady supply of stem cells within the meristems throughout active cell division cycles (Heidstra and Sabatini, 2014). In Arabidopsis, shoot apical meristem (SAM) can be divided into three regions: the central zone (CZ) at the apex of SAM, the peripheral zone (PZ) surrounding the CZ, and an internal ribbed meristem under the CZ. Within Arabidopsis PZ, the lateral primordia and consequent organs are generated in a Fibonacci spiral pattern, named phyllotaxis (Galvan-Ampudia et al., 2016). After transition from vegetative to reproductive growth, SAM is transformed into inflorescence meristem (IM), and then produces lateral floral primordia and organs.

The spatial distribution of the phytohormone auxin is mediated by numerous transmembrane efflux and influx carriers, and plays a crucial role in a wide variety of morphogenetic processes (Wang and Jiao, 2018). Among them, PIN-FORMED (PIN) family of auxin efflux carriers are localized in the plasma membrane on the same side of neighboring cells, and are important for the establishment and maintenance of morphogenetic auxin gradient (Adamowski and Friml, 2015). In SAM, the key PIN-family member PIN1 protein is expressed predominantly in the epidermis and provasculature (Heisler et al., 2005). The polar auxin transport mediated by PIN1 in SAM generates local auxin maxima and minima. Auxin maxima at the PZ are responsible for the specification and positioning of incipient primordia and associated lateral organs. In the mutant depleting of PIN1, the inflorescence apices are blocked in floral meristem initiation and displayed a pin-like naked stems (Reinhardt et al., 2000). Transcriptional regulation of *PIN1* has been considered to alter its protein abundance and enable regulatory cascade changes based on local auxin concentration (Reinhardt et al., 2003; Heisler et al., 2005; Habets and Offringa, 2014).

Auxin binding to the auxin receptor triggers the de-repression of downstream AUXIN-RESPONSE FACTORs (ARFs) implicated in auxin signaling (reviewed in Peer, 2013). Among them, ARF5 is a key transcription factor acting downstream of auxin perception (Reinhardt et al., 2000) and is critical for floral primordium initiation (Zhao et al., 2010). Intriguingly, *PIN1* transcription is also induced by auxin signaling through ARF5. Given the PIN1-dependent formation of auxin maxima, it may form a positive feedback that is of importance for the self-organization properties of the SAM (Wenzel et al., 2007; Krogan et al., 2016). ARF5 activates downstream genes highly expressed in organogenic regions of the reproductive shoot apex, such as *FILAMENTOUS FLOWERS* (*FIL*), and *TARGET OF MONOPTEROS 3* (*TMO3*) (Wu et al., 2015). It also represses downstream genes such as the two A-type *ARABIDOPSIS RESPONSE REGULATOR* (*ARR*) genes, *ARR7* and *ARR15*, which negatively regulate SAM size (Zhao et al., 2010).

In Arabidopsis primary roots, the maintenance of root apical meristem (RAM) requires two main parallel pathways. One is known as the *SHORT-ROOT* (*SHR*)/*SCARECROW* (*SCR*) pathway, two genes encoding the plant-specific GRAS family putative transcription factors (Helariutta et al., 2000; Sabatini et al., 2003). The other is the *PLETHORA1/2* (*PLT1/2*) pathway, which encode the AP2-class transcription factors (Aida et al., 2004; Blilou et al., 2005). *PLT1/2* genes are transcribed in response to auxin accumulation. Notably, members of *PIN*-family genes including *PIN1* collectively control the polar auxin distribution to determine the auxin maximum in RAM. Their combined action plays an important role in the expression pattern of *PLT* genes and further in stem cell specification.

Both chromatin-remodeling factors and histone chaperones can modulate local and global chromatin structure, playing crucial roles in DNA replication, transcription and repair (reviewed in Zhou et al., 2015; Ojolo et al., 2018). INOSITOL AUXOTROPHY 80 (INO80) is the founding member of the INO80 family chromatin-remodeling factors displaying diverse regulatory activities, such as nucleosome positioning and histone variant H2A.Z dynamics (reviewed in Gerhold and Gasser, 2014). In Arabidopsis, the *AtINO80* loss-of-function mutant *atino80-5* displays pleiotropic phenotypes including smaller organs and late flowering (Zhang et al., 2015). NAP1-RELATED PROTEIN (NRP) represents a highly conserved protein family of histone chaperones (reviewed in Zhou et al., 2015). Arabidopsis homologs *NRP1* and *NRP2* are functionally redundant, and their double mutant (*nrp1-1 nrp2-1*, abbreviated as *m56-1* in the previous study) displays short roots without any obvious phenotypes in the aerial organs (Zhu et al., 2006). Intriguingly, both *AtINO80* and *NRP1/2* are implicated in the frequency regulation of somatic homologous recombination (HR), which is an important pathway to repair DNA double-strand break (DSB), a lethal DNA damage if not repaired (Gao et al., 2012; Zhang et al., 2015). In our previous study, we generated the *atino80-5 m56-1* triple mutant, and observed a genetic epistasis of *m56-1* over *atino80-5* in the regulation of somatic HR frequency (Zhou et al., 2016). However, functional interactions between *AtINO80* and *NRP1/2* in the context of whole plant growth and development still remain largely obscure.

In this study, we report that *AtINO80* and *NRP1/2* synergistically control the proper floral primordia initiation and maintain the IM size. Transcription levels of several auxin-related genes were mis-regulated in the *atino80-5 m56-1* triple mutant. We showed the recruitment of AtINO80 and NRP1/2 as well as the decreased H3 occupancy in the chromatin regions of *PIN1*. In addition, *AtINO80* and *NRP1/2* concerted to prevent the cell death and DSB appearance in RAM and the accompanied activation of transcriptional response to DNA damage. These findings reveal their coordination in the maintenance of functional apical meristems.

## Materials and Methods

### Plant Materials and Growth Conditions

The wild-type (WT) and mutant lines *atino80-5* (Zhang et al., 2015) and *m56-1* (Zhu et al., 2006) are all derived from the Columbia (Col) ecotype background. The reporter lines *WOX5:GFP* (Blilou et al., 2005), *pPIN1:PIN1-GFP* (Benková et al., 2003) and *DR5rev:GFP* (Friml et al., 2003) in Col-background have been described in previous studies. Seedlings were grown vertically on agar-solidified MS medium M0255 (Duchefa) supplemented with 0.9% sucrose at 21°C under 16 h light/8 h dark conditions. For the inhibition of polar auxin transport, *N*-naphthylphthalamic acid (NPA, 33371, Sigma-Aldrich) was added to the medium at the indicated concentrations.

### Microscopy

The images of inflorescence were acquired by using a TM-3000 scanning electron microscope according to the manufacturer instructions (HITACHI). Differential interference contrast (DIC) images were taken with an Imager A2 microscope (Zeiss). For Lugol staining, roots were immersed in Lugol iodine solution containing 5% iodine for 2 min. After washing, roots were cleared with chloral hydrate solution (chloral hydrate: water: glycerol, 8:3:1, *w*/*v*/*v*). Confocal images were acquired by using a LSM710 microscope (Zeiss) with the following excitation/emission wavelengths: 561 nm/591–635 nm for Propidium Iodide (PI), 488 nm/505–530 nm for GFP. The antibody against γ-H2A.X was generated in our previous study (Zhou et al., 2016). The whole-mount root immunostaining was performed as previously described (Ma et al., 2018).

### Quantitative Reverse Transcription-Polymerase Chain Reaction (RT-PCR)

Plant organs were dissected by using a sharp blade and quickly frozen in liquid nitrogen. We used TRIzol kit to extract RNA according to standard procedures (Invitrogen). RT was performed using Improm-II reverse transcriptase (Promega). Quantitative RT-PCR was performed in three biological replicates. *ACTIN2* (*ACT2*) was used as a reference gene to normalize the data. The gene-specific primers are listed in the Supplementary Table 1.

### Chromatin Immuno-Precipitation (ChIP) Analysis

Chromatin immuno-precipitation was performed as described in our previous study (Zhang et al., 2015). All analysis was performed in three biological replicates. Antibodies used in this study were anti-GFP (A-11122, Invitrogen), anti-H2A.Z (Zhang et al., 2015), and anti-H3 (ab1791, Abcam). The gene-specific primers are listed in the Supplementary Table 1.

## Results

### The Triple Mutant *atino80-5 m56-1* Displays a Disordered Inflorescence Phenotype

**FIGURE 1:**
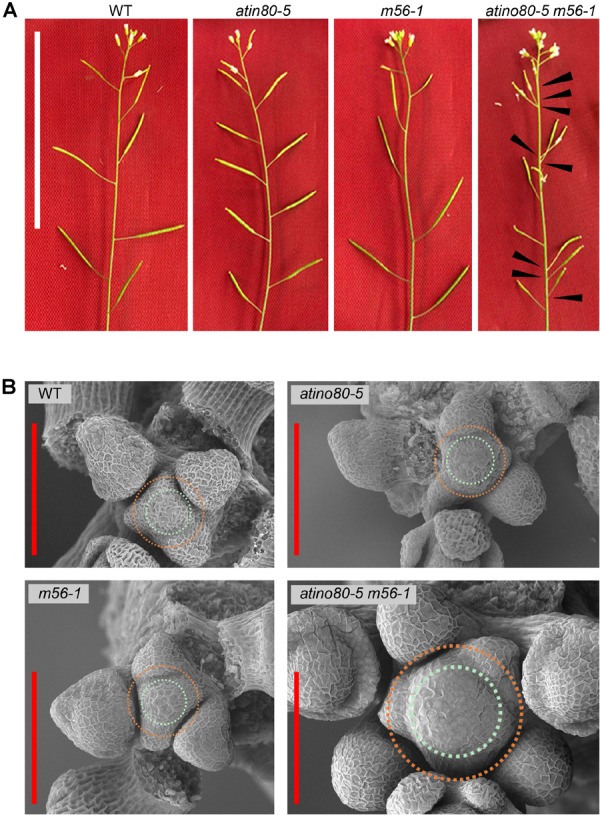
The disordered inflorescence in *atino80-5 m56-1* triple mutant. **(A)** Comparison of floral branches in WT, *atino80-5*, *m56-1* and triple mutant. Note that the spiral positioning of siliques was disrupted in triple mutant, which are marked by black arrowheads. Bar = 50 mm. **(B)** Scanning electron microscopy of IM in WT, *atino80-5*, *m56-1* and triple mutant. For visual comparison, the CZ in each IM is outlined with bluish circle, and the PZ is outlined with orange circle. Note that the IM size is significantly enlarged in triple mutant and more floral primordia were found in the same IM. Bar = 100 μm.

Our previous study has showed that the aerial part of *m56-1* double mutant seedling resembles that of WT, while *atino80-5 m56-1* triple mutant seedling resembles the single mutant *atino80-5* (Zhou et al., 2016). Here, we confirmed the maintenance of such epistatic effect on aerial growth throughout the whole vegetative stage. Except the decrease in leaf size observed for *atino80-5* and *atino80-5 m56-1*, no significant change of leaf phyllotaxy has been found in all the mutants (Supplementary Figure 1). After flowering, the WT flowers and siliques successively appeared along the branch axes in a Fibonacci spiral pattern. Such spiral pattern was not lost in *atino80-5* and *m56-1* inflorescences, albeit the spacing of *atino80-5* siliques was shortened (Figure 1A). Intriguingly, we found an obviously disordered positioning pattern of siliques along floral branches of the *atino80-5 m56-1* triple mutant. In many cases, several siliques appeared adjacent to each other without a spiral pattern. In addition, the development of most siliques and their fertility were greatly impaired in the triple mutant (Figure 1A). Notably, although the differentiation of flower organs was not generally affected in all the mutants, the organ size was reduced in *atino80-5* and more severely in *atino80-5 m56-1* (Supplementary Figure 2).

We observed and compared the IM by using electron microscopy (EM) (Figure 1B). There was no significant difference in IMs between WT, *atino80-5* and *m56-1*, in which several floral primordia locate in a spiral pattern around the periphery zone (PZ). However, in the *atino80-5 m56-1* triple mutant IM, the CZ significantly expanded but still with isotropy, and at the same time, extraordinary number of flower primordia at various growth stages emerged concurrently around the PZ, in line with the observed disordered inflorescence phyllotaxy. Our EM observation indicated that AtINO80 and NRP1/2 play a synergistic role in the maintenance of normal IM size as well as proper pattern of lateral organ initiation in IM.

### AtINO80 and NRP1/2 Modulate Chromatin Regions of *PIN1*

**FIGURE 2:**
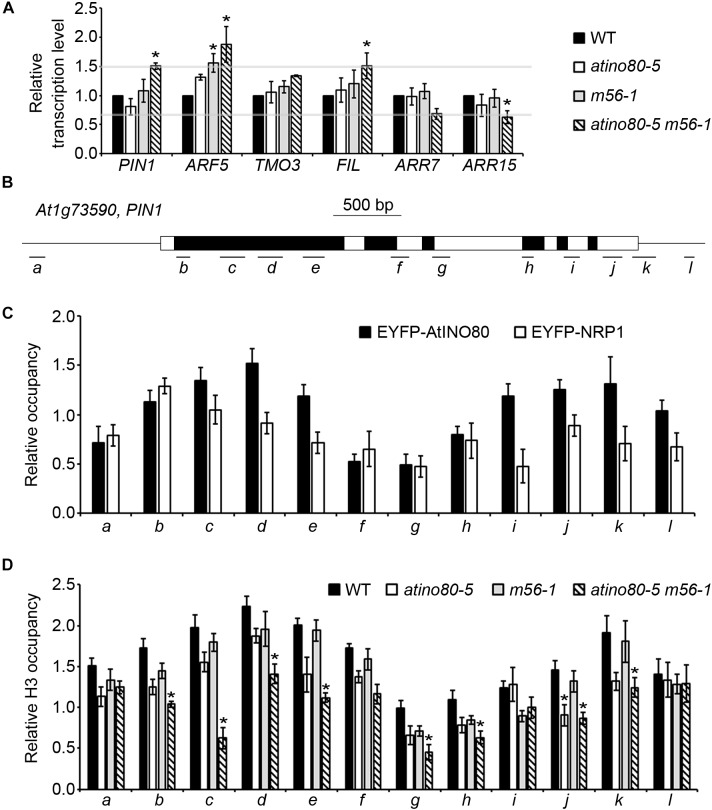
AtINO80 and NRP1/2 synergistically regulate *PIN1* transcription levels in inflorescences. **(A)** Relative transcription level of auxin-related genes in isolated inflorescences (>10 inflorescences as one replicate). *ACT2* was used as a reference gene. Relative values were further referenced to that of WT (set as 1). Mean values are shown with error bars from three independent replicates. Asterisks indicate statistically significant differences (*P* < 0.05, *t*-test) and fold change > 1.5 in mutants when compared with WT. **(B)** Schematic representation of *PIN1* gene structure. Black boxes represent exons; white boxes represent untranslated regions and introns; lines represent the promoter and terminator; letter-labeled bars represent regions amplified by the primer pairs that correspond to the letters on the *x*-axis of the underneath graphs. **(C)** Relative occupancy of EYFP-AtINO80 and EYFP-NRP1 in *PIN1* gene regions are revealed by ChIP using GFP antibody. Inflorescences of transgenic plants were collected for the ChIP analysis. *ACT2* was used as a reference gene. Mean values from three independent experiments are shown with error bars. **(D)** Relative occupancy of H3 in *PIN1* gene regions. Inflorescences of WT, *atino80-5*, *m56-1* and triple mutant were used for the ChIP analysis. *ACT2* was used as a reference gene. Mean values from three independent experiments are shown with error bars. Asterisks indicate statistically significant differences (*P* < 0.05, *t*-test) and fold change > 1.5 in mutants when compared with WT.

Both AtINO80 and NRP1/2 participate in local chromatin remodeling for transcription modulation (Zhang et al., 2015; Zhu et al., 2017). Given the vital role of auxin in determining floral primordia formation and in controlling IM size, we wonder whether auxin pathway is interrupted in the *atino80-5 m56-1* triple mutant. Therefore, we examined the transcription levels of several auxin-related genes in inflorescences. These include *PIN1*, *ARF5*, and the more downstream genes *FIL*, *TMO3*, *ARR7* and *ARR15*. Notably, the transcription levels of most examined genes are synergistically mis-regulated (fold change > 1.5) in the triple mutant (Figure 2A), in line with its growth abnormality in inflorescence phyllotaxy and SAM size.

In IM, PIN1 determines the polar distribution of auxin and triggers the consequent transcriptional cascade and organogenesis (Reinhardt et al., 2000). Hence, the roles of AtINO80 and NRP1/2 in *PIN1* transcriptional regulation were particularly examined in the following ChIP analysis by using inflorescences expressing EYFP-AtINO80 (Zhang et al., 2015) or EYFP-NRP1 (Zhu et al., 2017). Our ChIP results showed that EYFP-AtINO80 displayed enrichment at both 5′- and 3′-ends of the *PIN1* gene, while a single peak of EYFP-NRP1 was found after the transcription start site of *PIN1* (Figure 2B,C). These results indicated that *PIN1* is the target gene of chromatin-remodeling factor AtINO80 and histone chaperones NRP1/2, and at the same time suggested that the observed higher mRNA level of *PIN1* is not just the indirect result of enlarged IM size in the triple mutant inflorescence.

We also examined the occupancy of core histone H3 in WT and mutants in ChIP analysis. Relative H3 occupancy was slightly decreased in *atino80-5* and *m56-1*, but was clearly decreased (fold change > 1.5 and *P*-value < 0.05) in most examined regions of *PIN1* in the *atino80-5 m56-1* triple mutant when compared to WT (Figure 2D), which is consistent with the observed *PIN1* transcriptional change.

AtINO80 can regulate the local enrichment peak of histone variant H2A.Z within chromatin region of *FLC*, a key flowering suppressor gene (Zhang et al., 2015). Next, we analyzed the enrichment of H2A.Z relative to H3 (H2A.Z/H3) in *PIN1*. The H2A.Z/H3 peak was found near the 5′-end of *PIN1* in WT, which is largely maintained also in all the mutants (Supplementary Figure 3). The *atino80-5* mutant showed a reduction of H2A.Z/H3 but this reduction is compromised in *atino80-5 m56-1*, suggests that the H2A.Z dynamics is not associated with the synergistic effect of *atino80-5* and *m56-1* on the transcriptional up-regulation of *PIN1* in the *atino80-5 m56-1* triple mutant.

**FIGURE 3:**
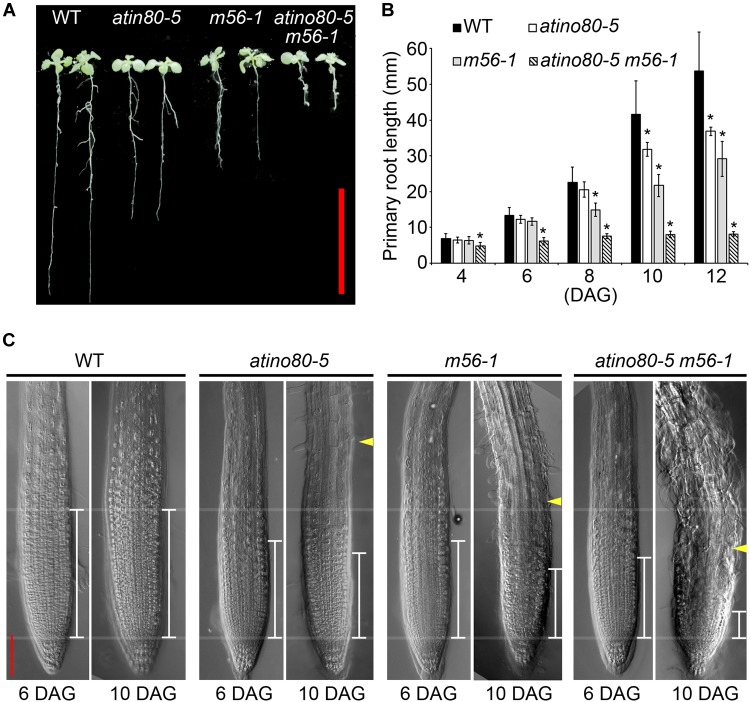
The short-root phenotype in triple mutants. **(A)** Primary roots in WT, *atino80-5*, *m56-1* and triple mutant at 12 DAG (days after germination). Bar = 20 mm. **(B)** Comparison of the primary root elongation in WT and mutants from 4 DAG to 12 DAG. Asterisks indicate statistically significant differences between the WT and mutants (*P* < 0.05, *t*-test). **(C)** Differential interference contrast (DIC) images taken on roots at 6 DAG and 10 DAG. The white scales mark the meristems, in which cells do not enlarge as revealed by DIC. The yellow arrowheads mark the root hair protrusion. Red bar = 100 mm.

### The Triple Mutant *atino80-5 m56-1* Exhibits Severe Root Growth Inhibition

Compared with *atino80-5* and *m56-1*, the triple mutant *atino80-5 m56-1* also displayed an additive short-root phenotype (Figure 3A). We measured the primary root elongation of vertically grown seedlings. The root length of *m56-1* became significantly shorter than that of WT from 8 day-after-germination (DAG), and that of *atino80-5* mutant became significantly shorter than WT from 10 DAG, which are consistent with our previous studies (Zhu et al., 2006; Zhang et al., 2015). Remarkably, as early as from 4 DAG, the triple mutant has already shown an obvious inhibition of root elongation and the synergistic effect between *atino80-5* and *m56-1* became evident along the time course of root growth analysis (Figure 3B).

We observed and compared the root tips through DIC microscopy (Figure 3C). At 6 DAG, although the root length of *atino80-5* and *m56-1* were comparable to WT, their meristem size was smaller than that of WT, and again, we observed a much smaller meristem size in the triple mutant roots. At 10 DAG, only the meristem in WT sustained the original size, whereas the corresponding size in all mutants gradually decreased when compared with their younger state. Among them, the change in the triple mutant was most severe.

### Skotomorphogenesis Is Epistatic to *AtINO80* and/or *NRP1/2* Depletion

Dark treatment (skotomorphogenesis) can cause a decrease in both *PIN1* transcription level and the shoot-to-root polar auxin transport in hypocotyl, resulting in auxin depletion in the RAM as well as the consequent reduced meristem size (Sassi et al., 2012). The skotomorphogenesis-associated mechanism seems to be compatible with the observed phenotype in triple mutant, thus prompting us to examine the mutants in dark treatment.

Under dark growth conditions, the hypocotyls of all the mutants elongated as those of WT (Supplementary Figure 4A). Moreover, dark treatment caused similar thinner roots and much smaller RAM in all the examined roots (Supplementary Figure 4B). These findings indicate that skotomorphogenesis is epistatic to *AtINO80* and/or *NRP1/2* depletion. Moreover, transcriptional analysis by using RNA extracted from hypocotyls revealed that *PIN1* transcription level remained at a basal level in all the hypocotyls grown in dark. After light exposure, *PIN1* was potently transcriptionally activated in hypocotyls in the triple mutant *atino80-5 m56-1* (Supplementary Figure 4C), consistent with the synergistic role of AtINO80 and NRP1/2 in *PIN1* transcriptional repression.

### Auxin Pathway Is Transcriptionally Affected in the *atino80-5 m56-1* Mutant Root Tips

**FIGURE 4:**
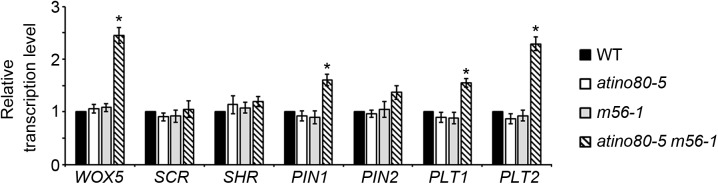
Transcription analysis of RAM-related genes in roots. Relative transcription level of RAM-related genes using roots at 10 DAG. *ACT2* was used as a reference gene. Relative values were further referenced to that of WT (set as 1). Mean values are shown with error bars from three independent experiments. Asterisks indicate statistically significant differences (*P* < 0.05, *t*-test) and fold change > 1.5 in mutants when compared with WT.

We further analyzed the transcription levels of several well-studied genes involved in RAM organization. They include: *WUSCHEL-RELATED HOMEOBOX 5* (*WOX5*), *SCR*, *SHR*, *PIN1*, *PIN2*, *PLT1* and *PLT2*. *WOX5* is a homeobox gene specifically expressed in quiescent center (QC) in RAM identity (Kong et al., 2015). *PIN2* encodes another PIN-family member which plays a root-specific role of auxin transport (Luschnig et al., 1998). Notably, the transcriptional levels of *WOX5*, *PIN1* and *PLT1/2* genes were synergistically and significantly up-regulated in the *atino80-5 m56-1* triple mutant (fold change > 1.5) (Figure 4), suggesting that auxin pathway also undergoes a transcriptional mis-regulation in the triple mutant roots.

Chromatin Immuno-Precipitation analysis by using roots as material was performed to examine whether the recruitment of AtINO80 and NRP1 in *PIN1* gene is consistent in different organs. Since transcription level of *NRP1* is lower than that of *NRP2* in Arabidopsis root (Supplementary Figure 5), we also introduced a transgenic plant expressing FLAG-NRP2 and included root-specific *PIN2* gene in the same ChIP analysis. The recruitments of EYFP-AtINO80 and EYFP-NRP1 in *PIN1* chromatin regions in roots was observed (Supplementary Figures 6A,B), with a pattern largely comparable to that previously described in inflorescences (Figure 2C), and the distribution pattern of FLAG-NRP2 was closely similar to that of EYFP-NRP1. In contrast, no obvious peaks of these proteins were found in *PIN2* chromatin regions (Supplementary Figures 7A,B). The pattern of relative H3 occupancies in *PIN1* was similar in roots with those in inflorescences (Supplementary Figure 6C). Meanwhile, reduction of relative H3 occupancy (fold change > 1.5 and *P*-value < 0.05) was found in some regions near the 5′-end of *PIN2* in *atino80-5 m56-1* triple mutant when compared to WT (Supplementary Figure 7C).

**FIGURE 5:**
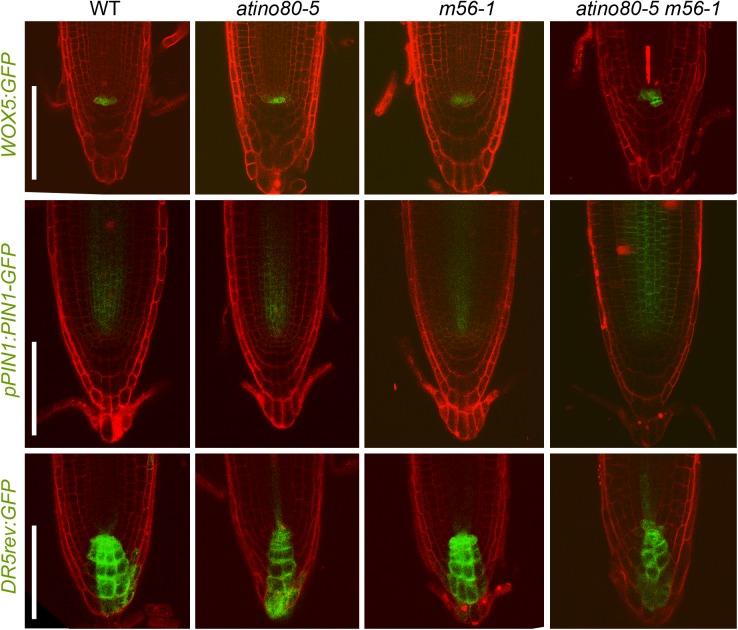
Expression patterns of several fluorescent reporters are affected in triple mutant. Expression patterns of *WOX5:GFP* (upper panel), *pPIN1:PIN1-GFP* (middle panel) and *DR5rev:GFP* (lower panel) in WT, *atino80-5*, *m56-1* and triple mutant at 6 DAG. PI staining was simultaneously used to label cell walls in root tips. Bar = 100 μm.

We also introgressed several fluorescent reporters including *WOX5:GFP* (Blilou et al., 2005), *pPIN1:PIN1-GFP* (Benková et al., 2003) and *DR5rev:GFP* (Friml et al., 2003) into each mutant background and observed their expression in root tips. A slightly stronger GFP signal of *WOX5:GFP* and *pPIN1:PIN1-GFP* were detected in the QC and steles in triple mutant, respectively (Figure 5, upper and middle panels). These findings are consistent with the above transcription analysis, and at the same time, also exclude the possibility that the severe short-root phenotype of triple mutant *atino80-5 m56-1* may be caused by the depletion of QC, which is crucial for the maintenance of stem cell niche (van den Berg et al., 1997).

In a good proportion of examined triple mutant roots (3 out of 10 samples), ectopic GFP signal of *WOX5:GFP* reporter was detected in the presumptive position of columella stem cells (Figure 5, upper panel). To verify the function of the columella cells, we also examined the root tips with Lugol solution and found the WT-like accumulation of starches in the columella cell layers in all the mutants (each *n* > 10) (Supplementary Figure 8), indicating that the differentiation of columella cells was not significantly impaired in the absence of AtINO80 or NRP1/2.

Fluorescent signal of *DR5rev:GFP* is located in QC/columella cells and enriched on the acropetal side as a polar gradient in WT. This gradient pattern was little affected in *atino80-5* and *m56-1* root tips, but was moderately interrupted in the columella cell layers in the *atino80-5 m56-1* triple mutant (Figure 5, lower panel), indicative of a disturbed auxin polar distribution.

### Triple Mutant *atino80-5 m56-1* Is More Sensitive to NPA Treatment

To get more insight into the auxin transport in the triple mutant root, we transferred 4-day-old vertically grown seedlings to the culture medium containing different concentration of *N*-naphthylphthalamic acid (NPA), a synthetic inhibitor of auxin transport. The presence of NPA inhibited the root elongation, and this inhibitory effect is NPA-concentration dependent (Supplementary Figure 9). We observed the inhibitory effect at different concentrations of NPA on RAM (Supplementary Figure 10). Under NPA treatment at high dosage (5 μM), the WT RAM was not significantly changed even when the root length has been strongly suppressed. In comparison, the RAM structure of *atino80-5* and *m56-1* were obviously altered: the root hairs were much closer to the tips, a defect largely similar to that observed in the *atino80-5 m56-1* triple mutant under untreated conditions. Notably, although treated with a low concentration of NPA (1 μM), the triple mutant root meristem displayed already an unusual expansion, which is accompanied by a quick differentiation of epidermal cells into root hairs (Supplementary Figure 10). Taken together, our observations indicate that the triple mutant roots are more sensitive to exogenous NPA treatment, providing additional evidences for its defects in maintaining functional auxin distribution in RAM.

### AtINO80 and NRP1/2 Synergistically Prevent Programmed Cell Death and γ-H2A.X Loci Accumulation in Root Tips

**FIGURE 6:**
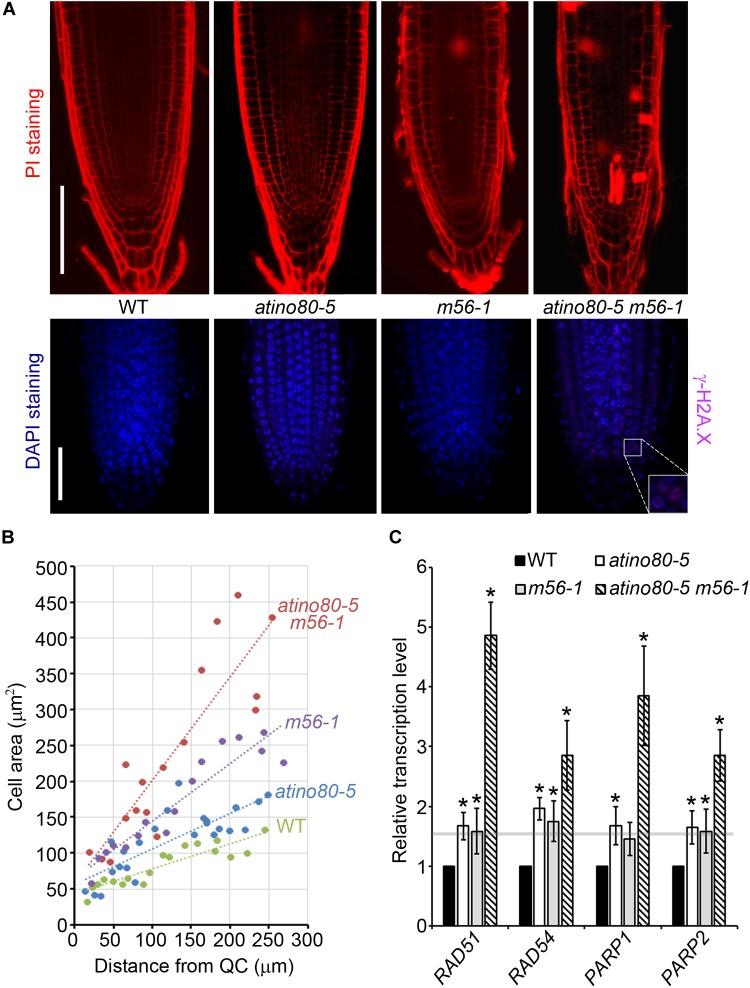
Genome instability in triple mutant. **(A)** (Upper panel) PI-stained root tips of WT, *atino80-5*, *m56-1* and triple mutant at 6 DAG. Bar = 100 μm. (Lower panel) Whole-mount root immunofluorescence staining analysis at 6 DAG. The γ-H2A.X signal detected using specific antibody is shown in pink and DNA staining by DAPI is shown in blue. Bar = 50 μm. **(B)** The increase of the cortical cell area (mm^2^) along with distance from QC cells. Regression lines are included. **(C)** Relative transcription level of DNA repair genes using roots at 6 DAG. *ACT2* was used as a reference gene. Relative values were further referenced to that of WT (set as 1). Mean values are shown with error bars from three independent experiments. Asterisks indicate statistically significant differences (*P* < 0.05, *t*-test) and fold change > 1.5 in mutants when compared with WT.

It has been reported that root stem cells and their early descendants can be selectively killed by genotoxic treatment causing DSB (Fulcher and Sablowski, 2009). PI staining can enter and mark dead cells because of the interrupted membrane integrity. We noticed that the *atino80-5 m56-1* triple mutant roots have accumulated PI-marked dead cells, which were barely found in WT or the *atino80-5* and *m56-1* mutant root tips (Figure 6A, upper panel). This observation suggests that AtINO80 and NRP1/2 synergistically prevent programmed cell death in root tips. H2A.X phosphorylation (γ-H2A.X) at the DNA break site constitutes one of the earliest events in the DNA repair process (Friesner et al., 2005). Although our previous study showed that the whole protein extracts from the triple mutant plants grown in the normal conditions did not show an obvious γ-H2A.X accumulation in Western blot analysis (Zhou et al., 2016), our immunostaining analysis detected weak but significantly visible γ-H2A.X loci in the root tips of the *atino80-5 m56-1* triple mutant (Figure 6A, lower panel).

Double-strand break can also induce the early onset of endoreduplication in cortical cells, which is frequently associated with cell enlargement (Adachi et al., 2011). A plot of cortical cell area against the distance from QC revealed that the cortical cell expansion were more pronounced in the triple mutant than in *atino80-5* and *m56-1* when compared to WT (Figure 6B). Furthermore, we examined transcription levels of DNA damage-sensory genes *PARP1/2* and DNA repair genes *RAD51/54*. All of these tested genes were synergistically up-regulated in the triple mutant roots (Figure 6C). Collectively, our data indicate that *AtINO80* and *NRP1/2* coordinate to maintain chromatin stability to prevent DNA damage for genome integrity.

## Discussion

On the basis of our previous study, we expanded our genetic analysis of *AtINO80* and *NRP1/2* in plant growth and development. Here, we report on the abnormal inflorescence and severe short-root phenotypes of the triple mutant *atino80-5 m56-1*. *AtINO80* and *NRP1/2* act synergistically to maintain the proper size of IM and to control the regular positioning pattern of floral primordia. Meanwhile, both factors act together to sustain the stem cell niche as well as functional auxin distribution in RAM. In particular, the triple mutant *atino80-5 m56-1* accumulates PI-marked dead cells and shows cortical cell enlargement in root tips, which are accompanied by the transcriptional activation of key DNA damage-sensory and damage-repair genes. These findings demonstrate that *AtINO80* and *NRP1/2* exhibit complex genetic interactions in the regulation of IM and RAM functions during plant development.

Within PZ of SAM, lateral organ initiation is determined by auxin maxima. The abnormal positioning of siliques and the disordered IM observed in *atino80-5 m56-1* imply a perturbation of auxin maxima in the mutant shoot apex. In agreement with this assumption, our RT-PCR analysis revealed an increased expression level of the auxin transporter gene *PIN1* in the *atino80-5 m56-1* triple mutant as compared to WT or to the single mutant *atino80-5* or to the double mutant *m56-1*. Although the underlying mechanism of auxin-triggered lateral organogenesis has been considered to be similar in the vegetative SAM and the reproductive IM (Wang and Jiao, 2018), the triple mutant *atino80-5 m56-1* did not show obvious lateral organ initiation defects at vegetative growth stage. One possible explanation to the absence of SAM defects but the presence of IM defects in the triple mutant is that IM may be more sensitive in auxin response than does SAM. In support of this idea, the single mutants *pin1* or *arf5* grows naked stalks without flowers but can still generate leaves (Przemeck et al., 1996). The vegetative SAM failed to form lateral leaf primordia only when *PIN1* and *ARF5* are simultaneously knocked out in the *pin1 arf5* double mutant (Schuetz et al., 2008). Alternatively, other possible explanation exists that the functional synergy of AtINO80 and NRP1/2 may be further integrated or redundant with other specific yet unknown pathways (or factors) in the organogenesis of vegetative SAM but not reproductive IM.

Both our RT-PCR and fluorescent reporter gene analyses further demonstrated up-regulation of *PIN1* and perturbed auxin maxima in the *atino80-5 m56-1* mutant roots. As the key factor in auxin transcription response, ARF5 directly interacts with the upstream regulatory region of *PIN1* and regulates the gene expression, forming an auxin gradient-trigged positive feedback in SAM self-organization (Krogan et al., 2016). Similar feedback mechanism is also used in RAM by PLT transcription factors (Blilou et al., 2005; Xu et al., 2006). In addition, more recent studies have revealed additional sequence-specific transcription factors targeting the *PIN1* gene, including MADS-domain transcription factor AGAMOUS-like 14 (AGL14) (Garay-Arroyo et al., 2013) and PIN2 PROMOTER BINDING PROTEIN 1 (PPP1), an evolutionary conserved plant-specific DNA binding protein (Benjamins et al., 2016). Currently, there is no evidence to support that AtINO80 and NRP1/2 possess any sequence-specific DNA-binding ability. Previously, NRP1 has been shown to interact with the MYB transcription factor WEREWOLF (WER) and to enrich at the WER-downstream gene *GLABRA2* (*GL2*), which encodes a homeodomain-leucine zipper transcription factor critical for root hair patterning (Zhu et al., 2017). Intriguingly, up-regulations of *ARF5* and *PLT1/2* were detected in *atino80-5 m56-1*, and enrichments of EYFP-AtINO80 and EYFP-NRP1 were observed at the *PIN1* locus. Whether AtINO80 and NRP1/2 are recruited to the *PIN1* locus through physical interaction with a specific transcription factor remains to be examined in the future.

BRAHMA (BRM), a SWI/SNF-family chromatin-remodeling factor (Clapier and Cairns, 2009), has been previously shown to play a role in Arabidopsis root development (Yang et al., 2015). Loss of function of *BRM* affected auxin distribution by reducing the transcription levels of several *PIN* genes as well as *PLT* genes. ChIP experiments showed that BRM can directly target the chromatin regions of several *PIN* genes including *PIN1* and activate their expression. BRM also antagonizes the function of Polycomb group (PcG) proteins, and down-regulates the repressive H3K27me3 chromatin mark within target genes (Yang et al., 2015). Here, our study on the synergy of AtINO80 and NRP1/2 provides evidence for the participation of chromatin-related factors other than BRM in epigenetic regulation of *PIN1*. Since the up-regulation of *PIN1* transcription in *atino80-5 m56-1* is opposite to the down-regulation of *PIN1* in the *brm* mutant, future genetic analysis will be needed to examine their functional crosstalk and epistasis, which is important for an increased comprehensive understanding of regulatory mechanisms in local transcription regulation implicated in auxin response.

Under normal growth condition, QC in the *atino80-5 m56-1* developing root tips at early stage is relatively intact, and starch normally accumulates in the columella cells. The interrupted auxin distribution could not fully explain the observed decay of RAM in the triple mutant. *AtINO80* and *NRP1/2* have been independently reported to participate in the maintenance of plant genome stability (Zhu et al., 2006; Zhang et al., 2015). Their genetic interplay has been analyzed in somatic HR and telomere length (Zhou et al., 2016). In this study, severe DNA damage was observed to accumulate in cells at the root tips of *atino80-5 m56-1*, as evidenced by the accumulation of γ-H2A.X loci and the activation of DNA damage sensory and repair genes. The PI-labeled dead cells and the accumulative cortical cell enlargement strongly point to the chromatin instability caused by *AtINO80* and *NRP1/2* depletion. It is reasonable to speculate that such chromatin instability contributes to the progressive exhaustion of normal stem cell niche and the aggravation of organ growth defects.

Our previous studies have examined the genetic interactions of *NRP1/2* with *FAS2* (Kaya et al., 2001), which encodes the second large subunit of the Arabidopsis histone chaperone Chromatin Assembly Factor-1 (CAF-1) complex (Gao et al., 2012; Ma et al., 2018). In the triple mutant *m56-1 fas2-4*, the lack of *NRP1/2* function aggravated the chromatin instability caused by the *FAS2* deletion and leads to disorganized stem cell niche, loss of stem cell identity, and constrained cell division in roots (Ma et al., 2018). We noticed some commonalities between *m56-1 fas2-4* and *atino80-5 m56-1*, such as combined gene function synergy in maintaining chromatin integrity and stability as well as growth of primary roots. Our ChIP analysis unraveled a decrease of histone H3 occupancy at *PIN1*, which is in line with the *PIN1* transcriptional activation, in the *atino80-5 m56-1* mutant. This observation may also be considered as a window reflecting defects of chromatin organization in the mutant. Previously, studies by using fluorescence *in situ* hybridization (FISH) and histone fusions with a fluorescent protein have demonstrated that histone exchange is dynamic and extensive chromatin reorganization occurs during cell differentiation in Arabidopsis roots (Costa and Shaw, 2006; Otero et al., 2016). CAF-1 plays a key function in chaperoning histone H3 during DNA replication, and consistently the *fas1* or *fas2* mutant exhibits severe defects in chromatin organization and function. In comparison, simultaneous loss of the H2A/H2B-type histone chaperones NRP1/2 and the ATP-dependent chromatin-remodeling factor INO80 in the *atino80-5 m56-1* mutant may also impact global chromatin organization and genome function.

During last few years, techniques in Arabidopsis have been developed for isolation of nuclei tagged in specific cell types (INTACT) by affinity purification based on expression of a biotinylated nuclear envelope protein in transgenic plants (Deal and Henikoff, 2011), and for genome-wide profiling of chromatin accessibility based on DNaseI digestion (DNase-seq; Zhang et al., 2012) or Tn5 transposase cleavage (ATAC-seq; Lu et al., 2017). ATAC-seq has been successfully coupled with INTACT to establish accessible chromatin landscape in root cells expressing a tag construct driven by the constitutive CaMV 35S promoter (Tannenbaum et al., 2018). In human cells, a nicking enzyme assisted sequencing (NicE-seq) has been reported for high-resolution open chromatin profiling on both native and formaldehyde-fixed cells (Ponnaluri et al., 2017). Future exploration of these different technologies and their application to our different mutants will provide invaluable insight about mechanisms of histone chaperones and chromatin-remodeling factors in regulating chromatin organization and root cell proliferation/differentiation.

## Author Contributions

HK performed the laboratory work and data analysis. JM set up the genetic introgression of transgenic markers and provided technical support in microscopy analysis. DW participated in the microscopy analysis. W-HS designed the experiments and revised the manuscript. YZ designed the experiments, performed the laboratory work and data analysis, and wrote the manuscript.

## Conflict of Interest Statement

The authors declare that the research was conducted in the absence of any commercial or financial relationships that could be construed as a potential conflict of interest.
