# Supplementary material for: Functional Coordination of the Chromatin-Remodeling Factor AtINO80 and the Histone Chaperones NRP1/2 in Inflorescence Meristem and Root Apical Meristem
Source: Front Plant Sci. 2019 Feb 7;10:115. doi: 10.3389/fpls.2019.00115 (PMC6374632; doi:10.3389/fpls.2019.00115)
Supplement: Supplementary file 1 [file Data_Sheet_1.pdf]

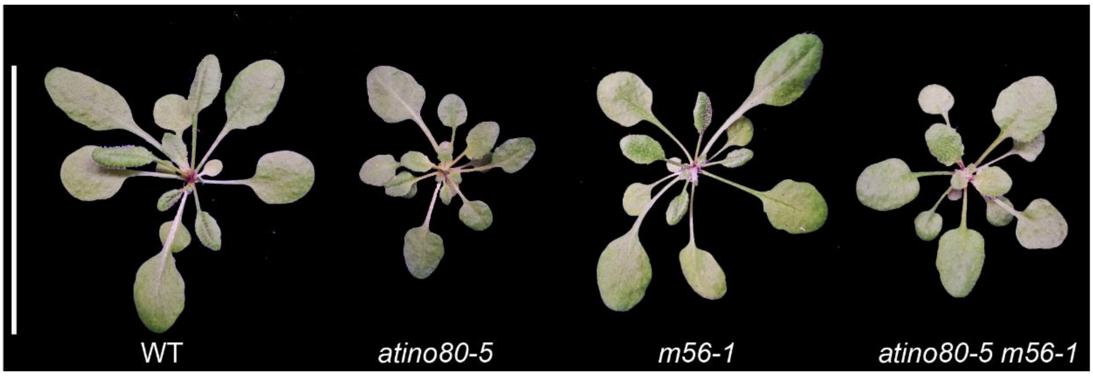

Supplementary Figure 1 | Leaf phyllotaxy is not obviously affected in all the mutants.

The rosette of WT, *atino80-5*, *m56-1* and triple mutant at 35 DAG were shown. Note that triple mutant are late-flowering as *atino80-5*, and WT and *m56-1* have flowered at this time. For visual comparison, the flowering shoots were removed from WT and *m56-1* plants. Bar=50 mm.

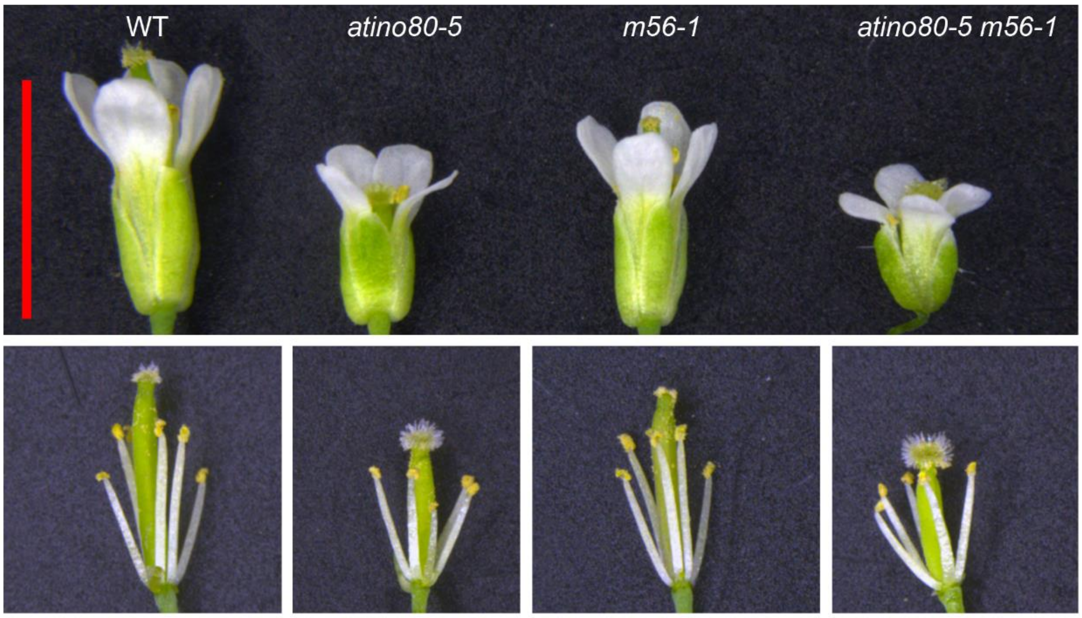

Supplementary Figure 2 | Flower organ size is significantly reduced in *atin80-5* single mutant and *atino80-5 m56-1* triple mutant.

Comparison of individual flower (upper panel) and stamen formation (lower panel) in WT, *atino80-5, m56-1* and triple mutant. Bar=50 mm.

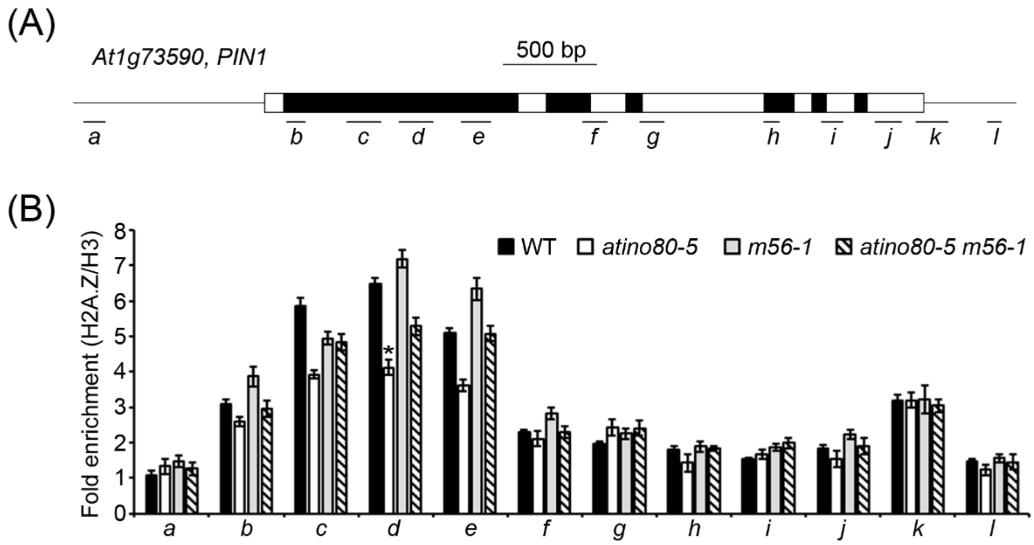

Supplementary Figure 3 | Fold enrichment of H2A.Z relative to H3 in chromatin regions of *PIN1*.

(A) Schematic representation of *PIN1* gene structure.

(B) Relative occupancy of H2A.Z relative to H3 (H2A.Z/H3) in *PIN1* chromatin regions. Inflorescences of WT, *atino80-5*, *m56-1* and triple mutant were used for the ChIP analysis. *ACT2* was used as reference gene. Mean values from three independent experiments are shown with error bars. Asterisk indicates both statistically significant differences ($P < 0.05$, *t*-test) and fold change > 1.5 in mutants when compared with WT.

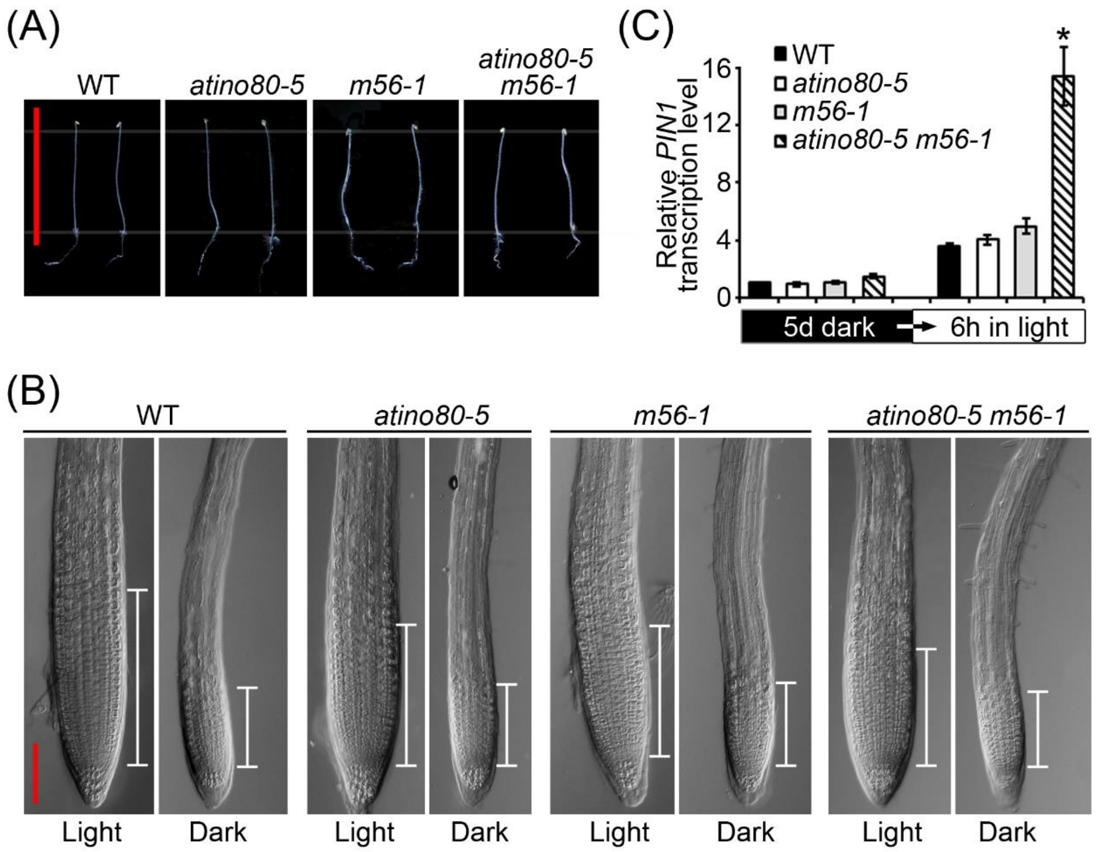

Supplementary Figure 4 | Skotomorphogenesis is epistatic to *AtINO80* and/or *NRP1/2* depletion.

(A) Comparison of etiolated seedlings at 5 DAG. Two lines were artificially added for visual comparison. Bar=100 μm.

(B) DIC images taken on roots grown in light or in darkness at 6 DAG. The white scales mark the meristem zone as revealed by DIC. Red bar=100 μm.

(C) Relative *PIN1* transcription level in hypocotyls in WT and mutants. Plants were first grown in darkness for 5 days and transferred to light growth conditions for further 6 hours. More than 20 hypocotyls were collected immediately after light exposure or after 6 hours of light treatment. *ACT2* was used as a reference gene. Mean value of *PIN1* transcription level of WT in darkness was set as 1. Mean values are shown with error bars from three independent experiments. Asterisks indicate statistically significant differences ($P < 0.05$, *t*-test) and fold change > 1.5 in mutants when compared with WT.

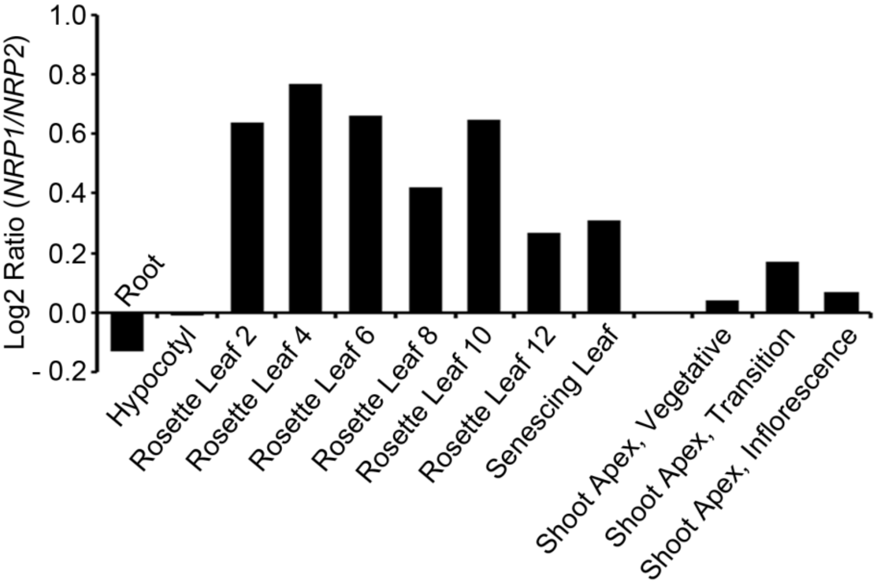

Supplementary Figure 5 | Relative transcription levels of *NRP1* and *NRP2*.

Expression values of *NRP1* (At1g74560, 260235_at) and *NRP2* (At1g18800, 261406_at) in diverse organs were extracted from Arabidopsis eFP Brower (http:// http://bar.utoronto.ca). The Data Source is “Developmental Map”. The Mode is “Compare”. Relative *NRP1/NRP2* expression values were shown in the form of Log2 Ratio. Note that *NRP1* transcription level is lower than *NRP2* in root, and is higher than *NRP2* in leafs and shoot apexes in all growth stages.

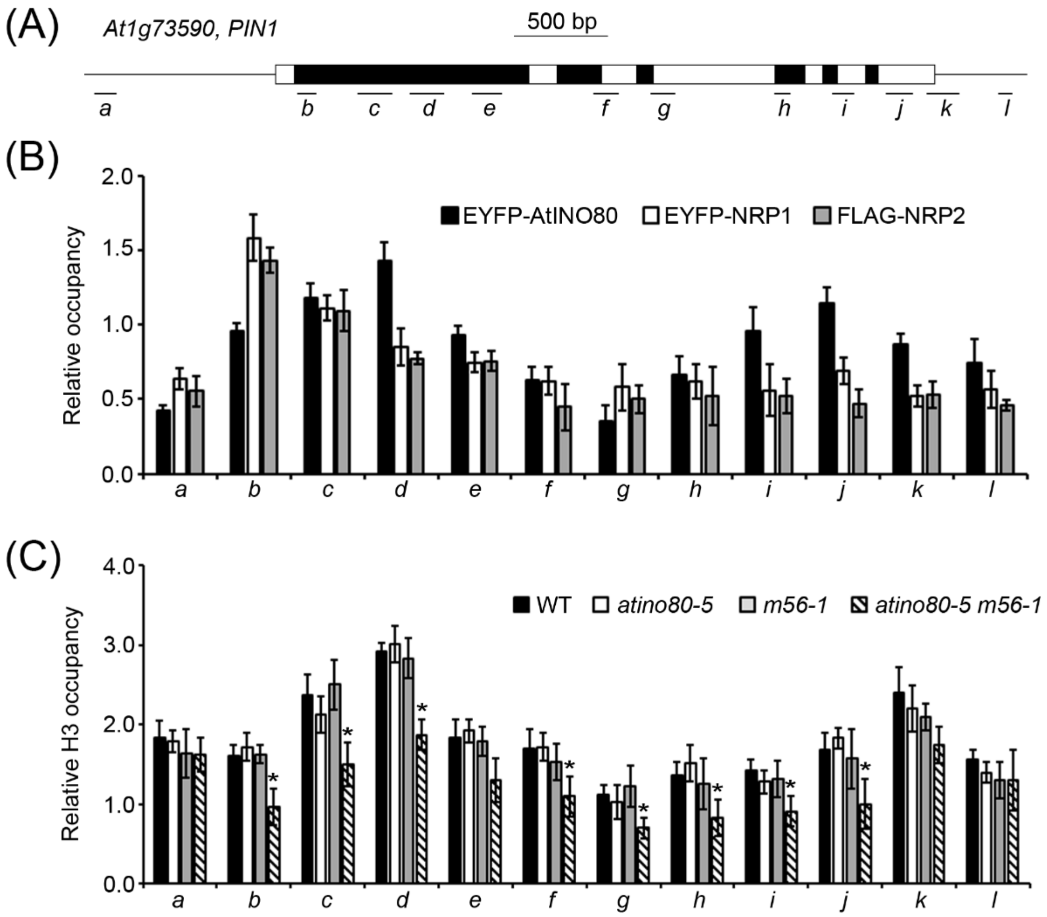

Supplementary Figure 6 | Recruitment of AtINO80 and NRP1/2 and relative occupancy of H3 in *PIN1* gene regions in roots.

(A) Schematic representation of *PIN1* gene structure.

(B) Relative occupancy of EYFP-AtINO80, EYFP-NRP1 and FLAG-NRP2 in *PIN1* gene regions are revealed by ChIP. Roots of transgenic plants were collected for the ChIP analysis. *ACT2* was used as a reference gene. Mean values from three independent experiments are shown with error bars.

(C) Relative occupancy of H3 in *PIN1* gene regions. Roots of WT, *atino80-5, m56-1* and triple mutant were used for the ChIP analysis. *ACT2* was used as a reference gene. Mean values from three independent experiments are shown with error bars. Asterisks indicate statistically significant differences ($P < 0.05$, *t*-test) and fold change > 1.5 in mutants when compared with WT.

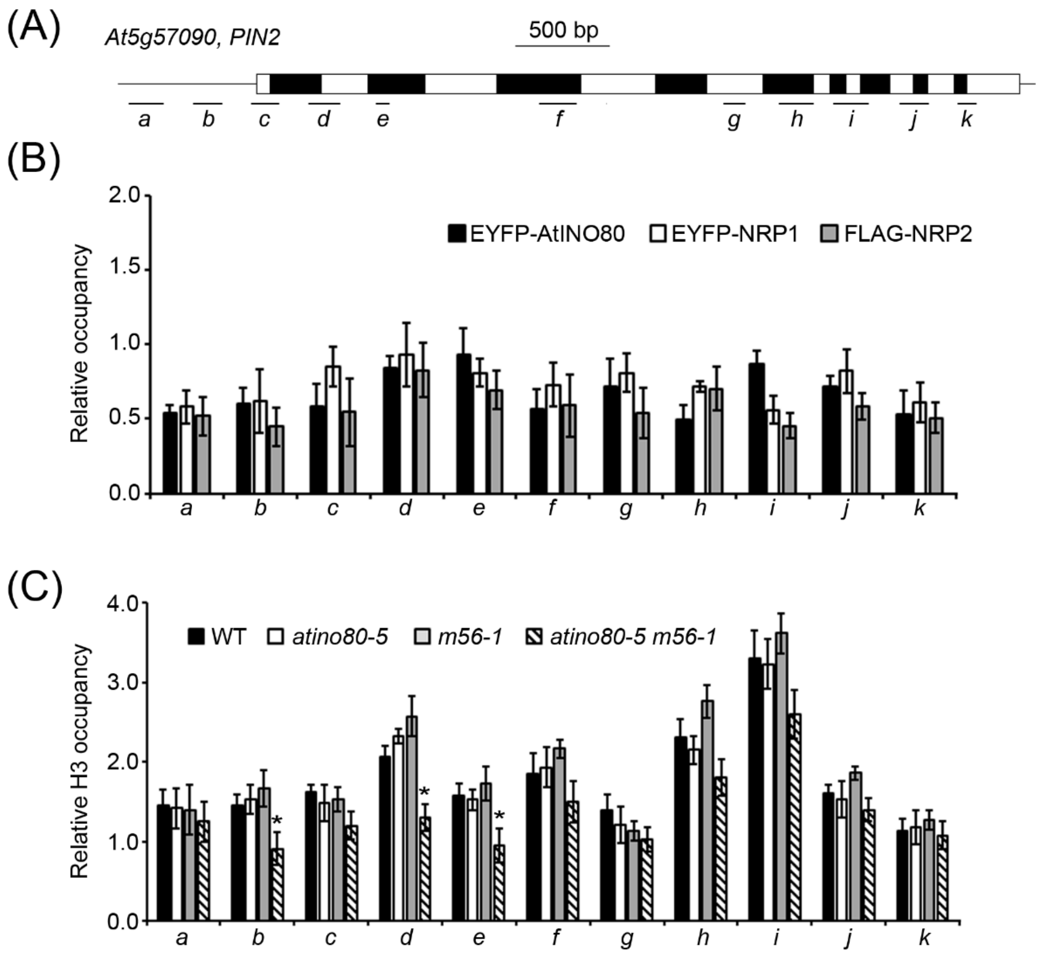

Supplementary Figure 7 | Recruitment of AtINO80 and NRP1/2 and relative occupancy of H3 in *PIN2* gene regions in roots.

(A) Schematic representation of *PIN2* gene structure.

(B) Relative occupancy of EYFP-AtINO80, EYFP-NRP1 and FLAG-NRP2 in *PIN2* gene regions are revealed by ChIP. Roots of transgenic plants were collected for the ChIP analysis. *ACT2* was used as a reference gene. Mean values from three independent experiments are shown with error bars.

(C) Relative occupancy of H3 in *PIN2* gene regions. Roots of WT, *atino80-5, m56-1* and triple mutant were used for the ChIP analysis. *ACT2* was used as a reference gene. Mean values from three independent experiments are shown with error bars. Asterisks indicate statistically significant differences (P < 0.05, t-test) and fold change > 1.5 in mutants when compared with WT.

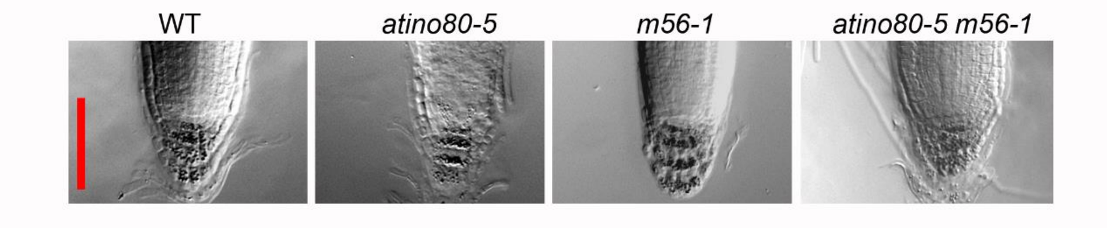

Supplementary Figure 8 | Differentiation of columella cells was not significantly impaired in triple mutant *atino80-5 m56-1*.

Accumulation of starch granules was revealed by Lugol staining at 6 DAG. Bar=100 μm.

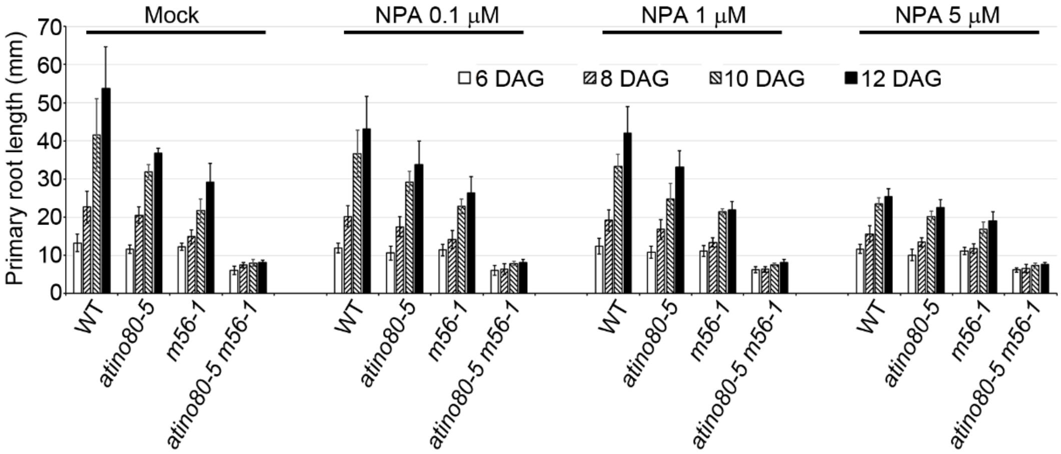

Supplementary Figure 9 | Primary root elongation is inhibited by NPA treatment.

4-day-old vertically grown seedlings were transferred to the culture medium containing indicated concentration of NPA for further cultivation. Primary root length was measured from 6 to 12 DAG.

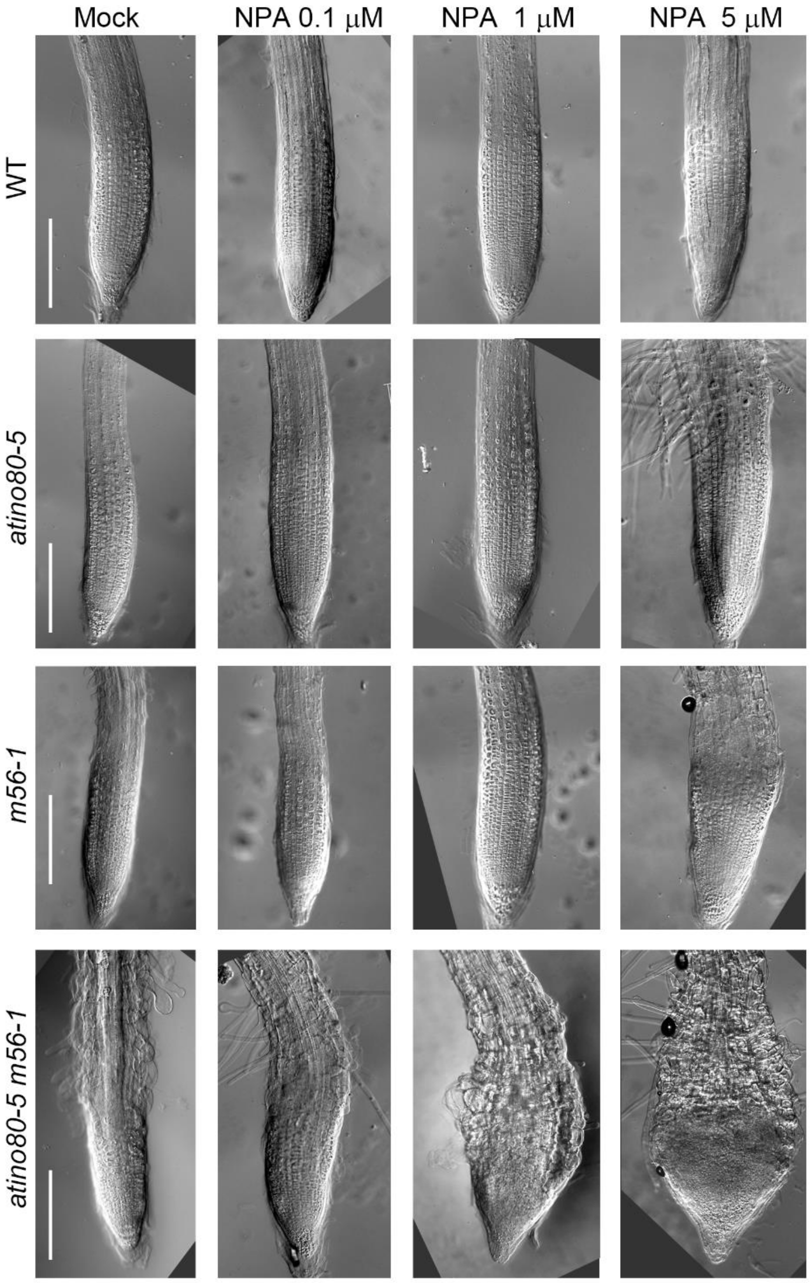

Supplementary Figure 10 | Triple mutant roots are more sensitive to NPA treatment.

4-day-old vertically grown seedlings were transferred to the culture medium containing indicated concentration of NPA for further 4-day cultivation. DIC images were then taken on root tips. Bar=200 μm.
