# Supplementary material for: Functional Coordination of the Chromatin-Remodeling Factor AtINO80 and the Histone Chaperones NRP1/2 in Inflorescence Meristem and Root Apical Meristem
Source: Front Plant Sci. 2019 Feb 7;10:115. doi: 10.3389/fpls.2019.00115 (PMC6374632; doi:10.3389/fpls.2019.00115)
Supplement: Supplementary file 2 [file Table_1.pdf]

Supplementary Table 1 | The primers used in this study.

For quantiative RT-PCR

		Sequence	T_m
AT1G73590 *PIN1*	PIN1-rF1	TGCCATTATCCAGGCAGCTT	59.74
	PIN1-rR1	AAGAGTTATGGGCAACGCGA	60.04
	Product length	130 bp	
AT5G57090 *PIN2*	PIN2-rF1	TTCTTTGGCAGGCGTTTAGC	59.4
	PIN2-rR1	ACCAACGTGTTAGGCAGTGT	59.82
	Product length	85 bp	
AT1G19850 *ARF5*	ARF5-rF1	TGGCTTCATTGTCTTGTGTTGA	58.71
	ARF5-rR1	CCGGCTTTCTTGTACCTGACT	60
	Product length	144 bp	
AT4G23750 *TMO3*	TMO3-rF1	CTCCTTTCTTCAACTTTCCGGG	59.19
	TMO3-rR1	GAAAAACACTGCGAGAGCCG	60.11
	Product length	72 bp	
AT2G45190 *FIL*	FIL-rF1	TCAGCTGTTTGTTCACCGGA	59.82
	FIL-rR1	GGAACATTAACCGCAAGGATGG	59.9
	Product length	95 bp	
AT1G19050 *ARR7*	ARR7-rF1	TCTCTTCTTGTAAAGTGACGACTGT	59.93
	ARR7-rR1	TTAGAAGCCCCTTTGCCTCC	59.67
	Product length	85 bp	
AT1G74890 *ARR15*	ARR15-rF1	AGAGTGGGACTAGGGCTCTG	60.03
	ARR15-rR1	CTGAGTGCTGAAGACTCTTTGA	58.01
	Product length	160 bp	
AT3G11260 *WOX5*	WOX5-rF1	AGAGGCAGAAACGTCGTAAA	57.19
	WOX5-rR1	CTTCGCTTATTTCAAAAACATCTCT	56.67
	Product length	87 bp	
AT3G54220 *SCR*	SCR-rF1	AAAGGGAAGCTGTGGCTGTT	60.11
	SCR-rR1	AGGAGCTAATCTTTGGAGTAACCA	59.22
	Product length	98 bp	
AT4G37650 *SHR*	SHR-rF1	ACGGAGCAATCTTGGAAGCA	59.96
	SHR-rR1	CTTGTTGGCCACGACAACT	58.6
	Product length	161 bp	
AT3G20840 *PLT1*	PLT1-rF1	AAGAGTTCGTGGCTGCCATT	60.25
	PLT1-rR1	CCATGTTGGTGATGCCTTGT	58.74
	Product length	91 bp	
AT1G51190 *PLT2*	PLT2-rF1	GGAACATTCAGCACGGAGGA	60.04
	PLT2-rR1	GTTGCTCTCCAGGATGGCTT	60.03
	Product length	129 bp	

For ChIP

	Sequence	T_m		Sequence	T_m
PIN1-a-F	AGAAAATAAGTTCCAAGATGAGCGA	58.83	PIN2-a-F	GTAAGATGCTCGCAAAAACCA	57.45
PIN1-a-R	TGAGATGGGTGTGTGTGAGTTAT	59.42	PIN2-a-R	CTTGGATCCACTTGTACGTGTAATA	58.67
Product length	118 bp		Product length	185 bp	
PIN1-b-F	CGGACTTCTACCACGTTAT	54.04	PIN2-b-F	AACGGCTTTTTGCAGAAGTAAT	57.42
PIN1-b-R	ATTGGTCTGGTGTGAAGATTT	55.38	PIN2-b-R	CTCGGCATTTTGTGAATGGAC	58.13
Product length	102 bp		Product length	155 bp	
PIN1-c-F	AACACTCTAGTCATGGGGAT	55.21	PIN2-c-F	ACTTCACTTCCTTGTCCATAAAT	55.83
PIN1-c-R	ACAATAGATCCTGCTGTGTC	54.86	PIN2-c-R	CACCATAGCCGCTAAAACATC	57.39
Product length	185 bp		Product length	149 bp	
PIN1-d-F	ACTGTTCGTCGTTCTAATGC	56.21	PIN2-d-F	ATTACCACTTCCTCGCTGC	57.54
PIN1-d-R	CAGAAGCCATCATCGAGTAAA	55.79	PIN2-d-R	GTTTTCGAGTAAAAATTGACCATC	55.32
Product length	184 bp		Product length	169 bp	
PIN1-e-F	TATCAATCTGGAGGAAGTGGT	55.76	PIN2-e-F	CTTTTCACTATCAACACTGCC	55.15
PIN1-e-R	GAAGATCTCTTCCGTTTCCG	55.98	PIN2-e-R	GAAGTCTCCGTACATCGCC	57.75
Product length	160 bp		Product length	73 bp	
PIN1-f-F	TACTCCAGTTTATTCGGCATC	55.49	PIN2-f-F	TACGCAATGTTTAACGCAAGC	58.42
PIN1-f-R	TCAAGGCTGACCCTTTATGC	57.87	PIN2-f-R	CACTCATACTTCTGCCTCCTC	57.55
Product length	135 bp		Product length	199 bp	
PIN1-g-F	TATGTTCAGTCTTGGTCAGTT	54.5	PIN2-g-F	AAAGCTCAATATCGGTTCTAAG	54.02
PIN1-g-R	AAAACACCATAAAGAATGAAGG	52.9	PIN2-g-R	CTTATAGAAAGCCGTACGGAAC	56.71
Product length	132 bp		Product length	117 bp	
PIN1-h-F	TAAAGGGTTGTTCATGGCGTTA	58.25	PIN2-h-F	GTCACCTTACATGGGCAAAAA	56.98
PIN1-h-R	CGACAAATCTCATAGCCGC	56.6	PIN2-h-R	TGAAAGAGACAAGGGACCAAG	57.51
Product length	86 bp		Product length	185 bp	
PIN1-i-F	AATGTGCATCCTGACATTCT	55	PIN2-i-F	CCAACGATAATGAGTGGATCG	56.59
PIN1-i-R	TTAATGTAGGCAAAACCAACCTA	56.09	PIN2-i-R	TTTCCGCACGCAATAATCTTT	57.16
Product length	118 bp		Product length	188 bp	
PIN1-j-F	TTGTGGGATGATGAATTGTGA	55.47	PIN2-j-F	GTTTTAGCTAGTGATGTTGGGT	56.35
PIN1-j-R	CTGAGCTCCTACTTAAGTCTCC	57.09	PIN2-j-R	CTTACGCAGTGCTGAGAATATC	57.04
Product length	144 bp		Product length	155 bp	
PIN1-k-F	AGCTTTGGCTACTTTTAGTGA	55.14	PIN2-k-F	GCTGGTTGCTTTGCCTGTAAC	60.6
PIN1-k-R	TCACATTTAGGCAAACGAAACA	57.1	PIN2-k-R	ACCTTTGGGTCGTATCGCCT	61.26
Product length	174 bp		Product length	101 bp	
PIN1-l-F	GAAGATAGGGGACTTGGTGC	57.67			
PIN1-l-R	AGATTCCACAAACGCAGTGA	57.75			
Product length	76 bp				
